# Paretic knee extensor strength, gait velocity, and fat mass are major determinants of peak aerobic capacity in subacute stroke: observational cohort study

**DOI:** 10.1038/s41598-020-70936-9

**Published:** 2020-08-18

**Authors:** Ji Hyun Kim, Eun Young Han, Sa-Yoon Kang, Sang Hee Im

**Affiliations:** 1 Department of Medicine, Graduate School, Jeju National University, Jejusi, Jejudo 63241 Republic of Korea; 2 Department of Rehabilitation Medicine, Jeju National University, College of Medicine, 15 Aran 13 gil, Jejusi, Jejudo 63241 Republic of Korea; 3 Department of Neurology, Jeju National University, College of Medicine, Jejusi, Jejudo 63241 Republic of Korea; 4 Department of Rehabilitation Medicine and Research Institute of Rehabilitation Medicine, Yonsei University College of Medicine, 50-1 Yonsei-ro, Seodaemun-gu, Seoul, 03722 Republic of Korea

**Keywords:** Stroke, Physical examination

## Abstract

The purpose of this study is to investigate major determinants of peak aerobic capacity in subacute stroke patients among body composition, balance function, walking capacity, and lower limb muscular strength. This was a retrospective observational cohort study. Eighty-three subacute stroke patients were enrolled and their medical records were retrospectively reviewed in the study (47 males; mean age: 62.95 ± 13.9 years). Gait capacity was assessed by gait velocity (10 m walk velocity:10MWV) and gait endurance (6 min walk distance:6MWD). Balance function was evaluated with Berg Balance Scale (BBS). The isometric muscular strengths of bilateral knee extensors were measured with an isokinetic dynamometer. Cardiovascular fitness was evaluated with an expired gas analyzer. In backward linear regression analyses, paretic isometric extensor strength (p < 0.001), fat mass (p = 0.005) and 10MWV (p < 0.001) are significantly correlated with peak aerobic capacity (adjusted R^2^ = 0.499) in all patients. Our results confirmed that paretic knee extensor strength, gait velocity, and fat mass were major determinants of peak aerobic capacity in subacute stroke. Therefore, therapeutic approaches should focus on improving gait velocity and paretic knee extensor strength in the early stages of recovery from stroke.

## Introduction

Stroke is one of the most common causes of death and, in survivors, often induces chronic residual deficits that impair walking ability and the basic activities of daily living (ADL)^1^. Thus, the goals of stroke rehabilitation therapy at the subacute stage is to enhance locomotion and basic ADL skills^2^. While 60–70% of subacute stroke patients can walk independently at discharge^3^, only 7% of stroke patients have sufficient capacity to maintain an independent gait in the community^3^. The barriers to community ambulation are related to persistent functional deficits such as muscle weakness, poor balance, and reduced mobility, which result in decreased physical activity and cardiorespiratory fitness^1^. Because cardiorespiratory fitness is significantly associated with baseline status and recovery of functional outcomes at the subacute stage, the reduced aerobic capacity might contribute to poor rehabilitation outcomes and exacerbate metabolic risk factors for recurrence of stroke, such as hypertension, obesity, and diabetes^4^. In addition, several studies have reported an association between body composition and cardiorespiratory fitness^5^. However, few studies in subacute stroke patients have comprehensively examined body composition and clinical functional outcome including gait performance, muscle strength, balance function, and cardiorespiratory fitness.

Therefore, the objectives of this study were to determine the major predictors of peak aerobic capacity in subacute stroke patients among body composition, balance function, walking capacity, and lower limb muscular strength.

## Methods

### Participant

This study was a retrospective observational cohort study. Eighty-three subacute stoke patients were recruited from October 2014 to July 2016 in department of rehabilitation medicine.

The inclusion criteria were described as; (1) A first-onset ischemic or hemorrhagic stroke patient that is confirmed by magnetic resonance imaging (MRI) or computed tomography (CT); (2) Disease duration within 3 months after onset;(3) Mild to moderate hemiparesis that is cable of walking at least 3 m, irrespective of a gait aid.

The exclusion criteria composed as below: (1) Severe deficits in communication, memory, or comprehension that cannot follow instructions or communicate with the investigators; (2) Cerebellar or brainstem lesions that demonstrates balance dysfunction; (3) Any additional neurological or orthopedic disease such as peripheral neuropathy, fractures, degenerative arthritis, or joint instability of hip or knee; (4) Psychiatric or cardiorespiratory problems; (5) Contraindication of maximal exercise testing, as proposed by the American College of Sports Medicine (ACSM)^6^.

All patients received conventional rehabilitation therapy composed of a 30-min of physical therapy and a 30-min of occupational therapy, twice per day, five times per week for 4 weeks. Retrospective chart review was performed to all patients according to medical history, physical and neurological examinations, and body composition. Given the retrospective study design, the need for informed consent was waived. Thus, the informed consent from patients in this research was waived by the institutional review board of Jeju National University Hospital (ethical approval number: (JNUH-IRB 2016-09).

### Bioelectrical impedance analysis and anthropometric measurements

Body composition data including body weight, fat mass, and muscle mass were measured using a bioelectrical impedance analysis (BIA) device (InBody520®, Biospace Co. Ltd, Seoul, Korea) on standing and gripping an eight-point tactile electrode system. 15 impedance values in five body segments were obtained by three distinct frequencies (5 kHz, 50 kHz, and 500 kHz)^7^. The body mass index (BMI) was then taken from (weight/height^2^) (kg/m^2^).

### Measurements of physical performance

#### Cardiovascular fitness: symptom-limited exercise tolerance test

Participants conducted a symptom-limited exercise tolerance test (ETT) by a treadmill (T-2100®, GE Healthcare Inc., Chalfont St. Giles, UK) or a cycle ergomety (Ergoselect 600 K®, Ergoline GmbH, Lindenstraße, Germany), with supervision of a physiatrist and a physical therapist.

After sitting for 15 min^8^, forty nine patients started to walk as fast as possible but safely on the treadmill at zero incline for the initial 2 min. After then, the workload was changed to a 4% incline for 2 min and increased by a 2% incline every 2 min. For thirty four patients that could not run on a treadmill, the cycle ergometry was alternatively used at a rate of 50–60 revolutions per minute (rpm) with a workload of 10 W for the first 2 min and the workload was raised by 5 W every 2 min. The test was terminated according to guidelines of ACSM^6^, if the patient asked or there was gait instability or signs of cardiovascular decompensation.

Peak exercise cardiovascular responses and maximal oxygen consumption (VO_2max_) by portable metabolic anaysis system (K4b2®, Cosmed, Rome, Italy) were obtained if at least two of the following five criteria for maximal effort were satisfied: (1) a respiratory exchange ratio (RER) ≥ 1.0;(2) a steady VO_2_ (< 150 mL/min) with increasing in exercise intensity;(3) no increase in heart rate (HR) despite workload increase;(4) % PHR > 95% of age-predicted maximal HR; or (5) volitional fatigue (a drop of cycling rate to < 30 rpm)^6^.

The parameters measured were peak oxygen consumption (VO_2peak_), resting/peak heart rate (RHR/PHR), resting/peak blood pressure (RBP/PBP), ETT duration, RER_peak_, and peak rate pressure product (RPP_peak_). RPP_peak_ was calculated as PHR × systolic PBP/100, and %PHR was converted from the percentage (%) of the age-predicted maximal HR [PHR /(220 – age)] × 100^6^.

#### Muscle isometric strength test: knee flexor and extensor strength

After 2-min rest, patients were measured the maximal isometric strength (torque, newton meter) of their knee extensor bilaterally using an isokinetic dynamometer (HUMAC NORM®, CSMI, Stoughton, MA, USA) on sitting with 85°-hip angles until the variation in torque was less than 5% for three successive trials^9^.

#### Gait performance

Gait performance was assessed by gait velocity (10 m walk velocity: 10MWV) and gait endurance (6-min walk distance: 6MWD). For 10MWV, subjects were instructed to walk 14 m (including both 2-m acceleration and deceleration phases) at a comfortable but fastest possible safe pace without verbal encouragement. Time was recorded in the use of a stopwatch and self-selected and fastest gait velocities (m/s) were calculated from the time taken to walk the middle 10 m track^10^.

For 6MWD, subjects were guided to walk as far as possible during 6 min, while walking through a 50 m hallway indicated with lines^11^.

#### Balance function: Korean version of Berg Balance Scale (BBS)

The BBS is a typical measurement of a subject’s balance and risk of fall which is composed of functional tasks common in everyday life^12^ and reveals high level of the inter-rater reliability (Kendall’s coefficient-0.97) and the intra- rater reliability in both physiatrist group (0.95) and physical therapist group (0.97)^13^.

The tasks progress from standing-up from a sitting position to tandem standing, and standing on one leg. Static and dynamic balance are evaluated by tasks such as reaching, standing position, and transference with a maximal score of 56 indicating good balance^14,15^.

### Statistical analysis

Descriptive analysis was used to characterize the samples and the distribution of variables. Average data are indicated as mean ± standard deviation (SD) for continuous variables including age, fat mass, muscle mass, BMI, and stroke duration, and all parameters of clinical outcome and cardiopulmonary fitness. χ^2^ tests were used for categorical variables. Pearson’s and spearmann correlation analysis was used to assess the relationships among clinical parameters with VO_2peak_, respectively. Pearson's and Spearmann’s correlation coefficients were categorized as below; weak < 0.3, 0.3 ≤ moderate < 0.7, strong ≥ 0.7^17^.

Multivariate linear regression analysis using backward selection linear regression model was employed to determine a significant predictor of cardiovascular fitness. All statistical analysis were performed using the SP statistical package ver. 20.0 (SPSS Inc.®,Chicago,IL, USA). A statistically significant difference was considered with a p-value of less than 0.05. Estimation of minimal sample size and statistical power was determined by G* Power program^16^.

### Ethical approval

All procedures performed in studies involving human participants were in accordance with the ethical standards of the institutional and/or national research committee (JNUH-IRB 2016–09) and with the 1964 Helsinki declaration and its later amendments or comparable ethical standards.

## Results

Baseline demographic and stroke-related data are presented in Table 1. Results for gait, balance, knee strength, and cardiovascular fitness for all patients are presented in Table 2. Mean values were listed as following: VO_2peak_ (18.62 ± 5.94 mL/kg/min); K-BBS score (39.87 ± 11.96); 10MWV (0.7 ± 0.39 m/s); 6MWD (200.95 ± 131.80 m); paretic and non-paretic knee extensor strength (56.93 ± 35.06 and 77.53 ± 35.6 Nm); paretic and non-paretic knee flexor strength (30.95 ± 20.93 and 44.46 ± 20.87 Nm).

**Table 1:** Demographic and disease-related characteristics of the subjects (*N* = 83).

Variable	Value
Age (years)	62.95 ± 13.9
Male	47 (56.6)
Height (cm)	161.94 ± 9.61
Weight (kg)	65.2 ± 11.48
BMI (kg/m^2^)	24.72 ± 2.94
Overweight	59 (71.1)
Muscle mass (kg)	43.59 ± 8.79
Fat mass (kg)	19.31 ± 7.58
Post-stroke duration (days)	32.54 ± 24.61
**Stroke type**	
Ischemic	59 (71.1)
Hemorrhagic	24 (28.9)
**Lesion side**	
Left	32 (38.6)
Right	51 (61.4)
**Comorbidities**	
Cardiovascular disease	4 (4.8)
Diabetes mellitus	19 (22.9)
Hypertension	48 (57.8)

Values represent mean ± standard deviation (SD) or number (%).

N, number, BMI, body mass index.

**Table 2:** The results of baseline evaluation of cardiovascular fitness, balance function, gait, and knee muscle strengths (*N* = 83).

Characteristic	Value
VO_2peak_ (mL/kg/min)	18.62 ± 5.94
RPP	20,154.37 ± 6,112.98
RSBP (mmHg)	123.00 ± 16.17
RDBP (mmHg)	75.98 ± 10.76
PSBP (mmHg)	160.75 ± 27.09
PDBP (mmHg)	81.78 ± 13.39
RHR (bpm)	84.13 ± 17.67
PHR (bpm)	123.73 ± 25.33
ETT duration (sec)	451.34 ± 223.31
RER	0.93 ± 0.10
K-BBS (score)	39.87 ± 11.96
10MWV (m/s)	0.70 ± 0.39
6MWD (m)	200.95 ± 131.8
**Knee extensor strength (Nm)**	
Paretic leg	56.93 ± 35.06
Non-paretic leg	77.53 ± 35.6
**Knee flexor strength (Nm)**	
Paretic leg	30.95 ± 20.93
Non-paretic leg	44.46 ± 20.87

Values represent mean ± standard deviation (SD).

N: number, VO_2peak_: peak oxygen consumption, RPP: rate pressure product, RSBP: peak systolic pressure, RDBP: peak diastolic pressure, PSBP: peak systolic pressure, PDBP: peak diastolic pressure, RHR: resting heart rate, PHR: peak heart rate, bpm: beats per minute, ETT duration: exercise treadmill testing time, RER: respiratory exchange ratio, K-BBS: Korean version of the Berg Balance Scale, 10MWV: 10-m walk velocity, 6MWD: 6-min walk distance, m/s: meter/second, m: meter, Nm: newton meter.

Using a priori determination of G* Power program, a minimal sample size of the backward multiple linear regression analysis with numbers of predictors, an α = 0.05, a statistical power of 80% and an assumed large effect of f^2^ = 0.35, was estimated as 57 and the range of critical r in the correlation analysis with an alpha = 0.05, a statistical power of 80% and an assumed moderate effect of H1 = 0.3, was calculated from − 0.214 to 0.214.

Peak aerobic capacity correlated significantly with muscle mass (*r* = 0.281, *p* = 0.011), fat mass (*r* = –0.382, *p* < 0.001), paretic knee extensor strength (*r* = 0.585, *p* < 0.001), non-paretic knee extensor strength (*r* = 0.398, *p* < 0.001), paretic knee flexor strength (*r* = 0.546, *p* < 0.001), non-paretic knee flexor strength (*r* = 0.431, *p* < 0.001), 6MWD (*r* = 0.532, *p* < 0.001), 10MWV (*r* = 0.563, *p* < 0.001), and BBS (*r* = 0.503, *p* < 0.001) (Table 3). And male gender is related to high peak aerobic capacity (*r* = 0.293, *p* = 0.007).

**Table 3:** Correlation between VO_2peak_ and body composition, knee muscle strengths, 6MWD, 10MWV, and K-BBS.

Variable	Correlation coefficient (*r*) VO_2peak_	*p*-value
Gender (male)	0.293*	0.007
Age (years)	− 0.121	0.274
BMI (kg/m^2^)	− 0.186	0.092
Muscle mass (kg)	0.281*	0.011
Fat mass (kg)	− 0.382**	< 0.001
Paretic knee extensor strength (Nm)	0.585**	< 0.001
Non-paretic knee extensor strength (Nm)	0.398**	< 0.001
Paretic knee flexor strength (Nm)	0.546**	< 0.001
Non-paretic knee flexor strength (Nm)	0.431**	< 0.001
6MWD (m)	0.532**	< 0.001
10MWV (m/s)	0.563**	< 0.001
K-BBS (score)	0.503**	< 0.001

*p < 0.05, **p < 0.01.

BMI: body mass index, 10MWV: 10-m walk velocity, 6MWD: 6-min walk distance, VO_2peak_: peak oxygen consumption, K-BBS: Korean version of the Berg Balance Scale, Nm: newton meter, m: meter, m/s: meter/second.

**Figure 1:**
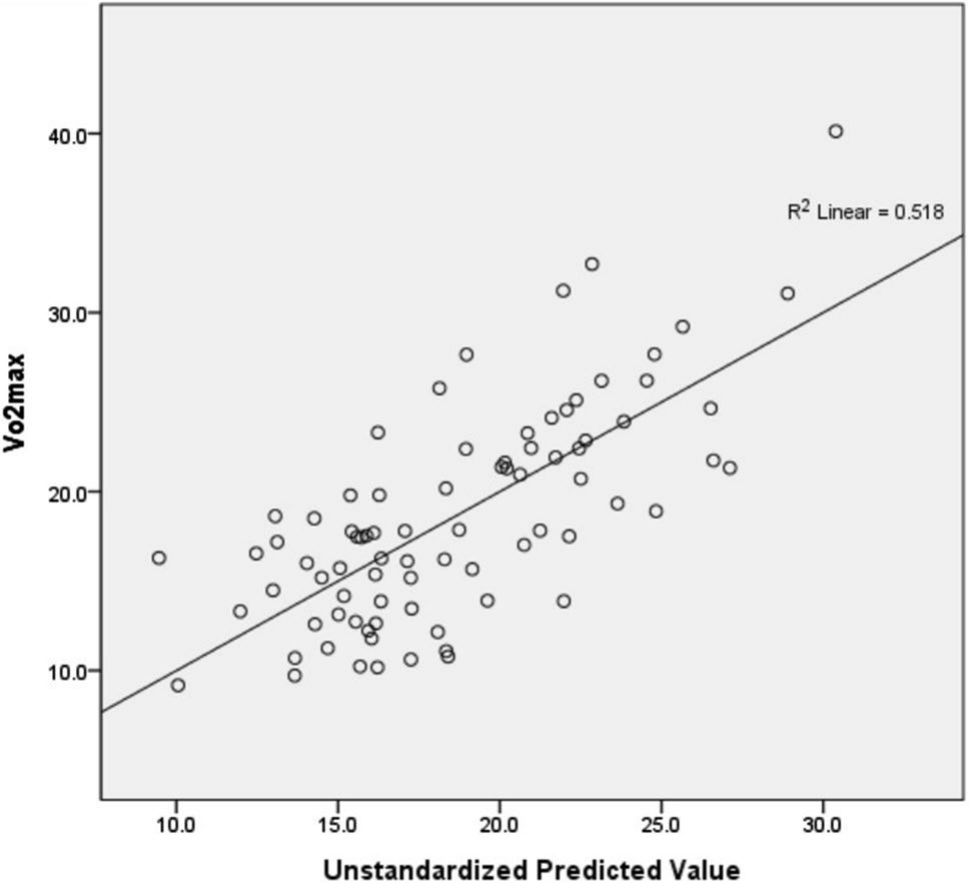
Scatter plots for multiple regression analysis.

In the backward linear regression analyses, paretic isometric extensor strength (*p* < 0.001), fat mass (*p* = 0.005), and 10MWV (*p* < 0.001) were independent predictors of peak aerobic capacity (adjusted *R*^2^ = 0.499) in all patients (Fig. 1).

## Discussion

In this study, we comprehensively evaluated cardiovascular fitness, lower limb strength, gait, and balance function in patients with subacute stroke and confirmed the close association of cardiorespiratory fitness with body composition and clinical functional outcomes.

Decreased aerobic fitness is common in stroke patients^18^. The mean VO_2peak_ in our participants was 18.82 ± 5.94 mL/kg/min, comparable to the low VO_2peak_ of 15.6 ± 5.5 ~ 19.7 ± 6.7 mL/kg/min reported in previous studies of patients with subacute stroke. Although few subjects met the criteria for maximal effort, they performed effectively in the symptom-limited exercise stress test, suggesting that the estimated values were reliable. Light basic ADLs require approximately 3 metabolic equivalents (METs) (10.5 mL/kg/min) of oxygen consumption and more intense ADLs require about 5 METs (17.5 mL/kg/min)^4^. Thus, the aerobic capacity of our patients was insufficient to perform various activities in the community. In addition, peak aerobic capacity was closely related to gait performance and lower limb muscle strength, also in agreement with previous finding^21^.

Our novel finding was that paretic knee extensor strength, gait speed, and fat mass were independent predictors of peak aerobic capacity in patients with subacute stroke. Patterson et al.^21^ previously showed that gait function influences cardiorespiratory fitness, and Baert et al. reported a relationship between VO_2peak_ and 6MWD in mild stroke patients^22^. However, our data indicate that gait velocity rather than gait endurance predicts cardiovascular fitness. Walking speed can be considered an easy and accessible indicator of independent walking in the community and Perry et al. found that gait velocity was highly related to different levels of ambulation^23^: household ambulation refers to a severe gait disorder with a walking velocity of less than 0.4 m/s; limited community and full community ambulation indicate moderate to mild gait disorders with walking speeds greater than 0.4 m/s and 0.8 m/s, respectively. Another study reported that a significant correlation between walking speed and community ambulation, and gait speed (> 0.66 m/s), could differentiate between community and non-community ambulators in stroke patients^24^. Because reduced gait speed may increase the energy cost of walking^25^, gait speed may be a key determinant of peak exercise capacity in stroke patients, especially those who are independent ambulators.

Lower limb weakness occurs frequently in stroke survivors, especially in the quadriceps and hamstring, which induces several kinematic gait problems^26^. Our previous results revealed that hemiparetic knee extensor strength is a predictor of gait endurance and that balance function is also a major determinant of gait speed and endurance in subacute stroke patients. During the stance phase of gait, the knee extensors support knee stability, thereby enabling the non-paretic limb to proceed in a swing phase during long-distance walking^20^. Furthermore, knee extensor weakness in stroke patients is caused by a reduction in recruitable motor units and a diminished capacity for oxidative metabolism^27^. Paretic lower limb strength is a strong predictor of gait function^28^, and Suzuki et al^29^. reported that knee muscle strength is significantly related to walking performance in stroke patients. Our findings also indicate that the peak torque of knee extensor strength is an independent predictor of cardiorespiratory fitness. Therefore, improving gait velocity and paretic leg extensor strength should be emphasized in patients with subacute stroke.

Exercise capacity is dependent on the severity of disease and body composition^30^. Our anthropometric analysis revealed that body-fat mass was inversely related to maximal aerobic capacity, whereas muscle mass and BMI were positively correlated with peak aerobic capacity. Individuals with abnormally high body-fat levels or a body weight exceeding the standard measure are defined as overweight (BMI ≥ 23) or obese (BMI ≥ 25)^31^. Of our participants, 71.1% (*N* = 53) were classed as overweight. Several studies have demonstrated a significant relationship between cardiorespiratory fitness and being overweight. Buchan et al. reported an independent association between cardiorespiratory fitness, waist circumference, BMI^32^ and Ortega et al. noted that cardiorespiratory fitness might have an effect on the association between being overweight and the level of physical activity^33^. The findings of these studies are consistent with the results of the present study; that is, there is a close relationship between body composition and aerobic capacity.

Most previous studies have assessed gait function using gait speed or short-distance walking only, but in this study we measured gait endurance and velocity, balance, body composition, and knee extensor strength in detail in a relatively large population of patients with subacute stroke.

However, this study had several limitations. First, we focused on knee muscle strength rather than hip and ankle muscle strength. We selected knee muscle strength because it is recovered early after stroke and is strongly correlated with gait function in patients with subacute stroke. The effect of muscle strength in different muscles may be researched in future studies. Second, the study was a cross-sectional study that focused on physical performance and we did not confirm the long-term effect on clinical or cardiorespiratory parameters. Third, in this study, cerebellum or brainstem infarction patients were not included. Because of, it is difficult to generalize to all stroke patients. Further studies should be conducted them. Finally, this study could not include patients with severe motor deficits who could not perform the ETT or gait tests. It is therefore difficult to generalize this result to all stroke patients.

In conclusion, this study presents scientific evidence for a significant association between cardiovascular fitness and body composition, gait performance, knee muscle strength, and balance function. Our results also show that gait speed, fat mass, and paretic knee extensor strength are major contributors to cardiovascular fitness in subacute stroke patients. Therefore, further studies focused on increasing muscle strength and gait speed and on managing fat mass might be warranted to improve cardiovascular fitness in subacute stroke rehabilitation therapy.
